# Cloning, ligand-binding, and temporal expression of ecdysteroid receptors in the diamondback moth, *Plutella xylostella*

**DOI:** 10.1186/1471-2199-13-32

**Published:** 2012-10-19

**Authors:** Baozhen Tang, Wei Dong, Pei Liang, Xuguo Zhou, Xiwu Gao

**Affiliations:** 1 Department of Entomology, China Agricultural University, Beijing 100193, China; 2 Inner Mongolia Prataculture Research Center, Chinese Academy of Sciences, Hohhot, 010031, China; 3 Department of Entomology, University of Kentucky, Lexington, KY 40546-0091, USA

**Keywords:** *Plutella xylostella*, Ecdysone receptor (EcR), Binding affinity, Expression profiling, Ecdysone agonist

## Abstract

**Background:**

The diamondback moth, *Plutella xylostella* (L.) (Lepidoptera: Plutellidae), is a devastating pest of cruciferous crops worldwide, and has developed resistance to a wide range of insecticides, including diacylhydrazine-based ecdysone agonists, a highly selective group of molt-accelerating biopesticides targeting the ecdysone receptors.

**Result:**

In this study, we cloned and characterized the ecdysone receptors from *P. xylostella*, including the two isoforms of EcR and a USP. Sequence comparison and phylogenetic analysis showed striking conservations among insect ecdysone receptors, especially between *P. xylostella* and other lepidopterans. The binding affinity of ecdysteroids to *in vitro-*translated receptor proteins indicated that PxEcRB isoform bound specifically to ponasterone A, and the binding affinity was enhanced by co-incubation with PxUSP (*K*_d_ =3.0±1.7 nM). In contrast, PxEcRA did not bind to ponasterone A, even in the presence of PxUSP. The expression of PxEcRB were consistently higher than that of PxEcRA across each and every developmental stage, while the pattern of PxUSP expression is more or less ubiquitous.

**Conclusions:**

Target site insensitivity, in which the altered binding of insecticides (ecdysone agonists) to their targets (ecdysone receptors) leads to an adaptive response (resistance), is one of the underlying mechanisms of diacylhydrazine resistance. Given the distinct differences at expression level and the ligand-binding capacity, we hypothesis that PxEcRB is the ecdysone receptor that controls the remodeling events during metamorphosis. More importantly, PxEcRB is the potential target site which is modified in the ecdysone agonist-resistant *P. xylostella.*

## Background

Ecdysteroids, a family of lipophilic poly-hydroxylated steroids, are hormonal factors modulating a diverse array of physiological processes such as development, reproduction, homeostasis and metabolism in arthropoda, especially in insects [1,2]. The proper developmental transitions in insects, including molting and metamorphosis [3], demand accurate cellular synthesis and reorganization and precise timing/programming of gene expression associated with various stages of development [4]. A classic example of such programming is reflected in the study of *Manduca sexta* (Lepidoptera: Sphigidae) by Lynn Riddiford and her colleagues [5,6]. In Lepidoptera, juvenile hormone (JH) is present rhythmically prior to the induction of ecdysteroids to orchestrate the precise developmental plan for insects [7]. 20-hydroxyecdysone (20E), is one of the most common and important ecdysteroids. The actions of 20E are evidently mediated by the ecdysone receptor complex, a heterodimer of two members of the nuclear receptors superfamily (NRs), the ecdysteroid receptor (EcR), and the ultraspiracle (USP) [8].

Structural features in these heterodimeric complexes shed lights on how ecdysteroids initiate molting and metamorphosis in insects. As reviewed by Nakagawa and Henrich [2], both EcR and USP exhibit a basic structure of typical NRs, commonly including 4-5 domains, namely the A/B, C, D, E, and in some receptors, F. The A/B domain at amino terminal is extremely variable, which contains a ligand-independent transcriptional activation function 1(AF-1), and interacts with other transcriptional factors. The C domain, the central DNA-binding domain (DBD), contains two highly conserved zinc finger motifs that are characteristic of the nuclear receptor superfamily (NRs). The D domain, a more variable region, is referred to as a hinge region between the C and E regions and harbors nuclear localization signals. It was reported by Graham *et al. *[9] that the D domain could influence the binding of ligand to the receptors in *Helicoverpa armigera*. The E domain, referred to as the ligand binding domain (LBD), functions uniquely to NRs and is well-conserved. For EcR, the LBD plays roles in receptor dimerization, ligand recognition and cofactor interactions. Recently, the flexible ligand-dependent binding pocket where steroidal and non-steroidal binds, has been characterized by X-ray crystallography [10-13]. The F domain is divergent and its functions are still unknown.

The diamondback moth, *Plutella xylostella* (L.) (Lepidoptera: Plutellidae), is a highly destructive pest of cruciferous crops worldwide, and has developed resistance to a wide range of insecticides, including the molt-accelerating compounds/ ecdysone agonists, such as diacylhydrazine (DAH) [14] insecticides [15,16]. DAH-based biopesticides have been used to control various agriculture, forestry, and stored product pests for the past decade [17-19], and been considered an environmentally friendly insecticide because of their remarkable selectivity across taxonomic orders, especially their compatibility with predatory biological control agents [20]. DAHs function by binding to the ecdysone receptor complex to compete with ecdysteroids, and to interfere with genes involved in the cuticle secretion to induce a lethal precocious incomplete molt, especially in Lepidoptera [21,22]. Previously we reported that the catabolism of ecdysteroid agonists (e.g., Fufenozide, a non-steroidal ecdysone agonist) may play a major role in the acquisition of fufenozide resistance in *P. xylostella*[23]. Other potential resistance mechanisms, such as the signaling of ecdysteroid receptor complex, involving both EcR and its heterodimer partner receptor USP [24], have yet been investigated.

As the eventual target of ecdysone agonists/molt-accelerating biopesticides, insect ecdysone receptors have been extensively studied in Diptera and Lepidoptera. In *P. xylostella*, an agriculturally important insect pest, ecdysone receptors have not been documented except EcRB (EF417582). In this study, we cloned and characterized the ecdysone receptors from *P. xylostella*, including both EcR and USP. The binding affinity of ecdysteroids to *in vitro -*translated receptor proteins was investigated. Moreover, the mRNA expression profiles of EcRs and USP, respectively, across different developmental stages were also documented. These combined results shed light on the molecular understanding of mechanisms underlying the ecdysone agonist resistance in *P. xylostella.*

## Methods

### Insects and RNA isolation

Larvae and adults of *P. xylostella* were maintained at 27 ± 1°C, 70 ± 10% RH, and a 16:8 L: D photoperiod, as described previously [23]. Total RNA was isolated from the whole body homogenates of the last-instar larvae (4^th^), pupae and adult females using TRIzol reagent (Invitrogen, Carlsbad CA, USA) following the manufacturer’s instructions. The concentration and purity of the total RNA were determined using a Thermo scientific NanoDrop 2000.

### Reverse transcription polymerase chain reaction (RT-PCR)

Reverse-transcription was conducted using PrimeScript 1st strand cDNA synthesize kit (Takara Biotechnology Co., Ltd, Dalian, China). For the cloning of *PxEcRB* (GenBank accession number: EF417582), specific primers (Additional file 1: Table S1) were designed and the PCR was performed with GC buffer Ι and LA Taq (Takara) as follows: 94°C/ 4 min; 30 cycles of 94°C /45 s, 57.8°C/40 s, 72°C/ 2 min; and 72°C/10 min. For *PxUSP*, degenerate primers (Additional file 1: Table S1) were designed based on amino acid sequences conserved in the C and E regions of other lepidopterean USPs, and the PCR was conducted as follows: 94°C/4 min; 30 cycles of 94°C/ 40s, 57.8°C/ 40s, 72°C/1 min20s; and 72°C/10min.

### Rapid amplification of cDNA ends (RACE)

Given that the isoform-specific A/B domains, the 5’-RACE primers for both *USP* and *EcR* were designed in the C region, and one reverse primer for 5’-RACE and one forward primer for 3’-RACE were designed, respectively (Additional file 1: Table S1). Total RNAs from adult females, pupae and the 4^th^ instar larvae, respectively, were subjected to 5’-RACE with Smart™ Race cDNA Amplification Kit (Clontech, Palo Alto CA, USA) according to manufacturer’s instructions. The cycles at annealing temperature of 68°C was 30 instead of 25. For 3’-RACE of USP, total RNA from the last-instar larvae was subjected to 3’-Full RACE Core Set Ver.2.0 (Takara) according to manufacturer’s instructions.

### Sequence analysis

PCR products were purified by agarose gel electrophoresis and cloned into the pGEM-T Easy vector (Promega, Madison WI, USA) before submission to Invitrogen (Shanghai, China) for sequencing. cDNA sequence, deduced amino acid sequences, and multiple sequence alignments were analyzed using DNAMAN 5.2 program. Sequence similarity of each domain imbedded in EcR and USP, respectively, was calculated by BLAST. Phylogenetic relationships of ecdysone receptors from *P. xylostella* with other insects were analyzed using CLUSTAL X 2.0 [25] and MEGA 5.0 [26] based on their amino acid sequences. Both NJ (neighbor-joining, model: poisson-correction, bootstrap values: 1000 replicates) and ML (maximum likelihood, model: Jones Taylor Thornton (JTT), bootstrap values: 500 replicates) trees were constructed and compared. All protein sequences were acquired from the GenBank.

### *In vitro* transcription-translation

Complete opening reading frames (ORFs) of *PxEcRA*, *PxEcRB* and *PxUSP* were amplified using primers listed in Additional file 1: Table S1 with LA Taq and cloned, respectively, into pF25 T7 Flexi Vector (Promega), which can act as an acceptor of a protein-coding region flanked by *Sgf*I and *Pme*I sites. *In vitro* transcription-translation of these constructs was carried out using T_N_T T7 Insect Cell Extract Protein Expression system (Promega) according to manufacturer’s protocol. To validate the expression, proteins were synthesized in the presence of Transcend™ tRNA (a precharged, ε-labeled biotinylated lysine-tRNA complex, Promega) and separated on a 10% SDS–PAGE gel. After electro-blotting, the biotinylated proteins were visualized by binding Streptavidin-Alkaline Phosphatase, and followed by the colorimetric detection.

### Ligand-binding assay

To compare the binding properties of the two EcR isoforms and to investigate their interactions with USP, the radioactive ligand-binding assay was performed according to Minakuchi *et al*. and Graham *et al*., respectively [9,27]. *In vitro* translated proteins were diluted 1:2 with low-salt buffer (20 mM HEPES, 20 mM NaCl, 10% glycerol, 1 mM EDTA, 1 mM 2-mercaptoethanol, pH 7.9, containing 1μg·ml^-1^ of aprotinin, pepstatin, leupeptin and 0.5 mg·ml^-1^ of bovine serum albumin). Diluted proteins (4 μl EcR, USP or a mixture of both) were incubated with 10 nM ^3^H-Labeled ponasterone A (tritiated PonA, 120 Ci·mmol^-1^, PerkinElmer Inc. Shelton CA, USA) in silicon tubes for 90 min at 25°C. No heterodimer protein controls (*in vitro* translation reaction prepared with an empty expression vector rather than a receptor) were regarded as the non-specific binding. The total volume of the reaction mixture was 16 μl, and the final concentration of solvent (ethanol) was less than 1%. At the end of the incubation, the samples were placed on ice and filtered immediately through nitrocellulose membrane (NC45, Merk Milipore) under a vacuum filtration apparatus. The membrane was then washed 3 times with 3ml of ice-cold washing buffer (low salt buffer with no protease inhibitors and BSA). Air-dried for ~10s, the membrane was transferred into a glass vial containing 2 ml of Aquasol-2 (PerkinElmer Inc.) in an oscillator for solvating. The radioactivity was then measured using a liquid scintillation counter Hidex-300SL (2 min/filter, Hidex instrument, Finland). In saturation binding experiments, proteins were incubated with 5 concentrations of tritiated PonA (0.625, 1.25, 2.5, 5.0 and 10 nM). Assays were performed in duplicate. The equilibrium dissociation constant (*K*_d_) and maximum binding capacity (*B*_max_) were caculated through non-linear regression using SigmaPlot 11.0 (Systat Software Inc., San Jose CA, USA).

### Quantitative real-time PCR (qRT-PCR)

Gene expression profiles of *PxEcRs* and *PxUSP,* respectively, were examined throughout the fourth instar and in different developmental stages. For expressions within the fourth instar, the time of newly exuviated fourth instar was assigned as 0 h and RNA samples were collected at 6 h intervals untill pre-pupation. For expression profiles across different developmental stages, the total RNAs were isolated from a mixtures of individuals developed at different time intervals, i.e., the RNA samples for the 3rd instar larvae were collected at 0, 12, 24 and 36h. Besides, adult samples were kept in male to female ratio of 1:1. Total RNA was extracted as described above and treated with DNase I (Takara), and then 1μg of total RNA was subjected to the PrimeScript 1st strand cDNA synthesize kit (Takara). The qRT–PCR was conducted using Platinum SYBR Green qPCR SuperMix-UDG Kit (Invitrogen) with a 20 μl reaction volume containing 250 nM primer and 100ng of cDNA in an ABI 7300 System. Ribosomal protein L32 of *P. xylostella* (PxL32) was used as a reference gene [28], and specific primers (Additional file 1: Table S1) for receptors were designed in the isoform-specific A/B domain using a web-based primer design platform, Primer 3 ( http://frodo.wi.mit.edu/primer3/). Standard curves of each gene were prepared, respectively, via serial dilutions (10×) of cDNA samples. The optimized qPCR profile was as follows: 50°C/2min, 95°C/2min, 50 cycles of 95°C/ 15s, 60°C/ 30s, and with a dissociation step. All calculations were carried out by the accompanying softwares of ABI 7300 System, and statistical analysis was conducted by one-way ANOVA and Tukey’s test using GraphPad InStat (GraphPad Software Inc., San Diego CA, USA).

## Results

### Molecular cloning of EcR and USP

**Figure 1:**
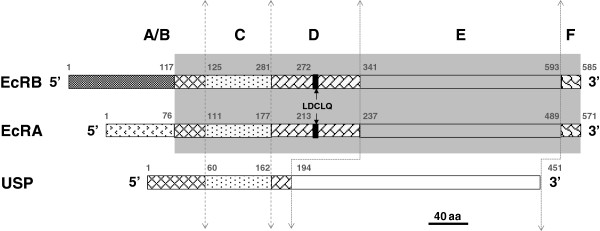
**Schematic drawings of PxEcR and PxUSP.A**/**B**, **C**, **D**, **E** ,and **F** domains are presented by rectangles of different forms. Conserved regions are highlighted in grey shade. The five amino acids (LDCLQ) in the D domain shown by black rectangle is absent in some cDNAs.

Using RT-PCR and 5’/3’ RACE, *PxEcRA* and *PxUSP* (GenBank accession number: ADA61199) were isolated. The sequenced *PxEcRA* open reading frame (ORF) is 1543bp in length and corresponds to a predicted protein with 512 amino acids (57KDa) (Additional file 2: Figure S1). The ORF of *PxUSP* is 1252bp in length and corresponds to a predicted protein with 415 amino acid residues (47KDa) (Additional file 3: Figure S2). For *PxEcRB*, a shorter ORF which lacks 15bp corresponding to 5 amino acids of LDCLQ in the D region in comparison to an existing GenBank entry (accession number: EF41758) was obtained (Figure 1 and Additional file 2: Figure S1). Both *PxEcRA* and *PxEcRB* have two homologous splicing variants in the hinge (D) region. The predicted proteins have typical structural characteristics of a nuclear receptor superfamily, including zinc finger motifs in the DNA binding region and ligand binding domain helices (Additional file 2: Figure S1 and Additional file 3: Figure S2), which further confirmed their identity as either EcR or USP homologs.

**Figure 2:**
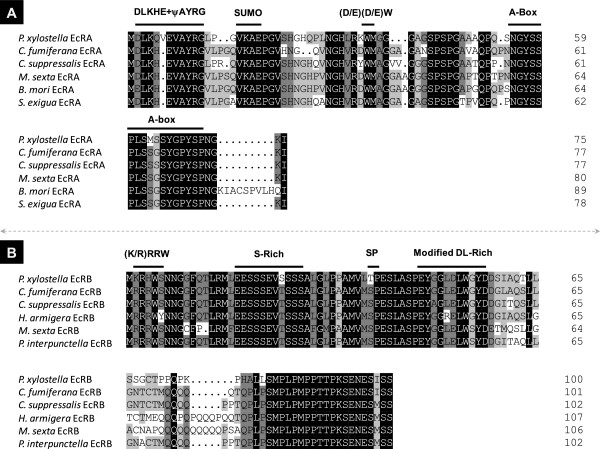
**Phylogenetic analysis of insect ecdysone receptors.** The phylogenetic relationships of *P. xylostella* ecdysone receptors, including EcRA (**A**), EcRB (**B**) and USP (**C**), with other insects were inferred using the NJ (neighbor-joining) estimation (poisson-correction model, 1000 bootstrap replicates).

All available insect ECRs and USPs from GenBank (as of December of 2011) were used for the phylogenetic analysis. The topology of both NJ and ML trees was very similar (Figure 2 and Additional file 4: Figure S3), therefore, only NJ tree (Figure 2) was presented here. It is not surprising that the full-length sequences of *PxEcRs* and *PxUSP* are most homologous to Lepidoptera, especially to the spruce budworm, *Choristoneura fumiferana* (Clemens), which shares 87%, 86% and 89% amino acid sequence similarity with PxEcRA, PxEcRB and PxUSP, respectively (Additional file 5: Table S2). EcRs and USP from lepidopteran insects were clustered together with high bootstrap supports (Figure 2). Furthermore, the sequence similarity of A/B, C, D and E regions of EcRs and USPs between *P. xylostella* and other insects were compared. The sequence similarity in the C region is very high (89-100%), especially the P- and D-box in the C region (Additional file 2: Figure S1 and Additional file 3: Figure S2), which are related to binding specificity of nuclear receptors to hormone response elements [29], are highly conserved. The sequence similarity of EcRs in the E region is also high: 82-95% for Lepidoptera, 68-73% for Diptera, and 63-64% for Coleoptera, respectively. The A/B and D regions are rather diverse among Lepidoptera. Similarly, the A/B, D and E regions of PxUSP are highly homologous to other lepidopterans. Overall, the phylogenetic relationships inferred from ecdysone receptors are consistent with the existing taxonomic relations among insects.

**Figure 3:**
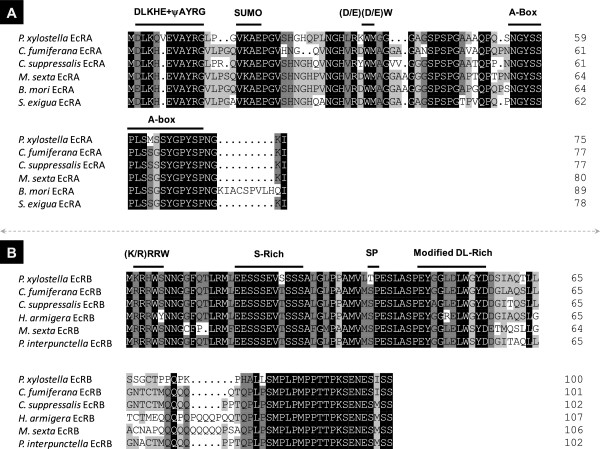
**Sequence alignment of EcR A/B domain. A**: Conserved motifs in PxEcRA include a Type 2 isoform-A specific box, two conserved N-terminal sequences (DLKHE and ΨAYRG), SUMOylation motif, and (D/E)(D/E)W residues. **B**: Conserved motifs in PxEcRB include a Type 6 isoform-B1 specific box, (K/R)RRW motif, S-rich motif, SP residues, and a modified DL-rich motif.

PxEcRA contains a conserved Type 2 isoform-A specific box, NGYSSP(M/L)SSGSYDPYSP [30], two conserved N-terminal sequences (DLKHE and ΨAYRG, where Ψ represents a large hydrophobic amino acid), the SUMOylation motif (small ubiquitin-related modifier ligases), and the (D/E) (D/E) W residues (Figure 3A). In contrast, PxEcRB has a Type 6 isoform-B1 specific box, which contains conserved microdomains (Figure 3B), including the (K/R) RRW motif, the S-rich motif (EESSSEVTSSS), the SP residues and the modified DL-rich motif. The sequence KREEKKA in the D region of PxEcR (Additional file 2: Figure S1) shows a putative nuclear localization signal (NLS). NLS has been identified as regions in which basic amino acids arginine and lysine are rich, and the NLS of PxUSP, homologous to *Drosophila melanogaster*, was found in the C region (Additional file 3: Figure S2).

### *In vitro* translation of PxEcRs and PxUSP

**Figure 4:**
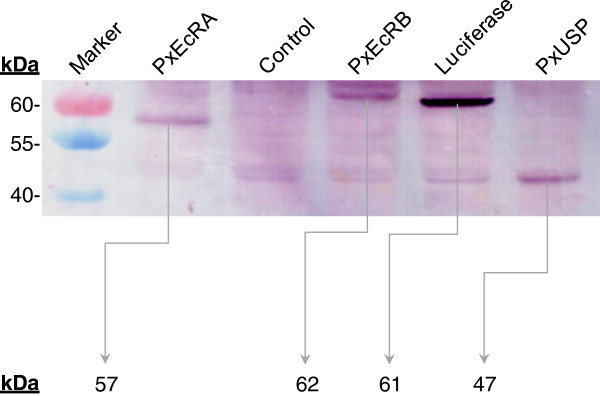
***In vitro *transcription-translation of *P. xylostella *ecdysone receptors.** Receptors were cloned into pF25 T7 Flexi Vector and expressed using T_N_T T7 Insect Cell Extract Protein Expression system. Proteins were synthesized in the presence of Transcend™ tRNA, separated on a 10% SDS–PAGE gel, and visualized by the colorimetric detection using streptavidin-alkaline phosphatase. EcRs, USP, Luciferase (positive control), and a negative control were visualized with/without gel band of the predicted size (PxEcRA: 57kDa, PxEcRB: 62kDa, PxUSP: 47kDa, Luciferase: 61kDa, respectively).

The two PxEcR isoforms and PxUSP were cloned into pF25 T7 Flexi Vector, respectively, and translated using T_N_T T7 Insect Cell Extract Protein Expression system, in the presence of Transcend™ tRNA. Polyacrylamide gel electrophoresis of biotinylated proteins revealed that the molecular weight of these proteins was in accordance with their predicted sizes (Figure 4).

### Specific binding of tritiated PonA to *in vitro*-translated protein

**Figure 5:**
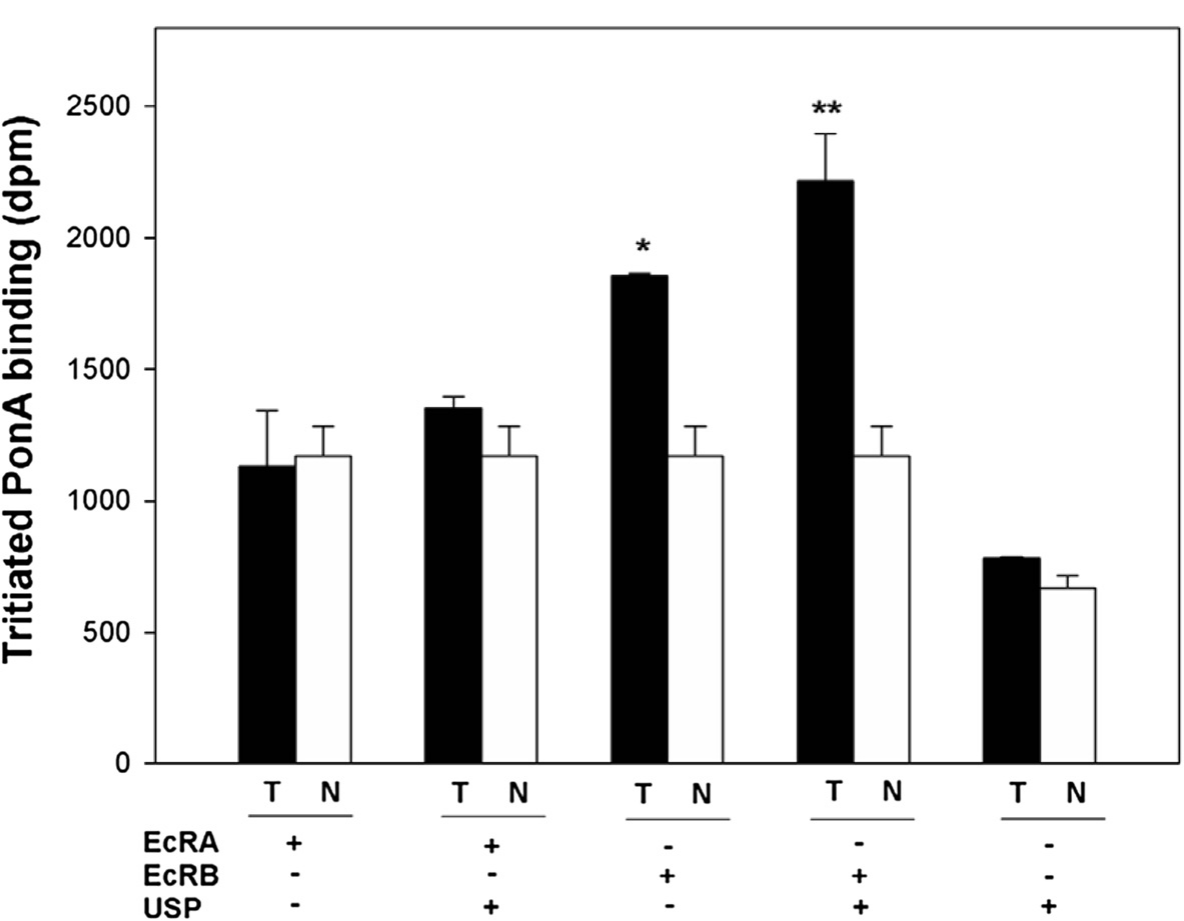
**Binding of tritiated ponasterone A to *in vitro-*translated EcRs and USP of *P. xylostella*, respectively.** Expressed proteins were incubated with 10nM tritiated PonA, and filtered through a nitrocellulose membrane. T, total binding; N, non-specific binding. The data were analyzed by one-way ANOVA and Tukey’s test using GraghPad InStat (GraphPad software Inc., San Digeo CA, USA). “*” and “**” denote that the total binding are significantly different with non-specific binding at α=0.05 and α=0.01 level, respectively.

**Figure 6:**
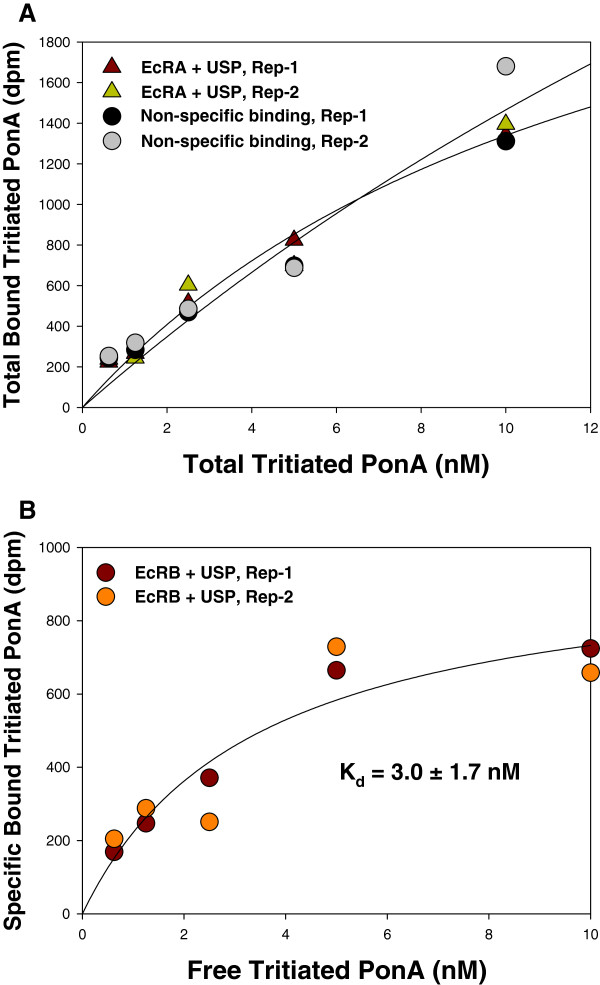
**Equilibrium binding of tritiated PonA to*in vitro*-translated EcRs/USP complex of*P. xylostella*.** Protein complex was incubated with different concentrations of tritiated PonA. Non-specific binding was determined using an expression vector. **A**: Total binding of EcRA+USP. **B**: Specific binding of EcRB+USP, as was calculated by subtracting the non-specific binding from the total binding. Non-linear curve fitting was plotted through SigmaPlot 11.0 (Systat Software Inc., San Jose, CA). Correlation coefficients (R) for the fitted curves were 0.979(non-specific), 0.987(EcRA+USP), and 0.943(EcRB+USP), respectively.

Tritiated PonA did not bind to PxEcRA and PxUSP (total binding was not greater than non-specific binding), but bond to PxEcRB specifically (Figure 5). Meanwhile, PxEcRB exhibited enhanced binding in the presence of PxUSP (specific binding for PxEcRB and PxEcRB/USP complex was 502 dpm and 1044 dpm, respectively). In contrast to PxEcRB, the co-incubation with PxUSP did not increase specific binding of PxEcRA (Figure 5), that is, the total binding was not greater than non-specific binding. Similar results were also found in saturation binding experiments, the total binding of PxEcRA/USP complex was equivalent to non-specific binding in each concentration of tritiated PonA (Figure 6A). As stated above, tritiated PonA bond specially to PxEcRB alone and to the PxEcRB/USP complex. Thus, in further saturation binding experiments, the dissociation equilibrium constant (*K*_d_) for the binding of tritiated PonA to PxEcRB/USP complex was calculated from the saturation curve of specific binding. The *K*_d_ value for PxEcRB/USP complex was 3.0±1.7 nM (Figure 6B), while the *B*_max_ was 963±221 dpm.

### Developmental expression profiles of PxEcRs and PxUSP

**Figure 7:**
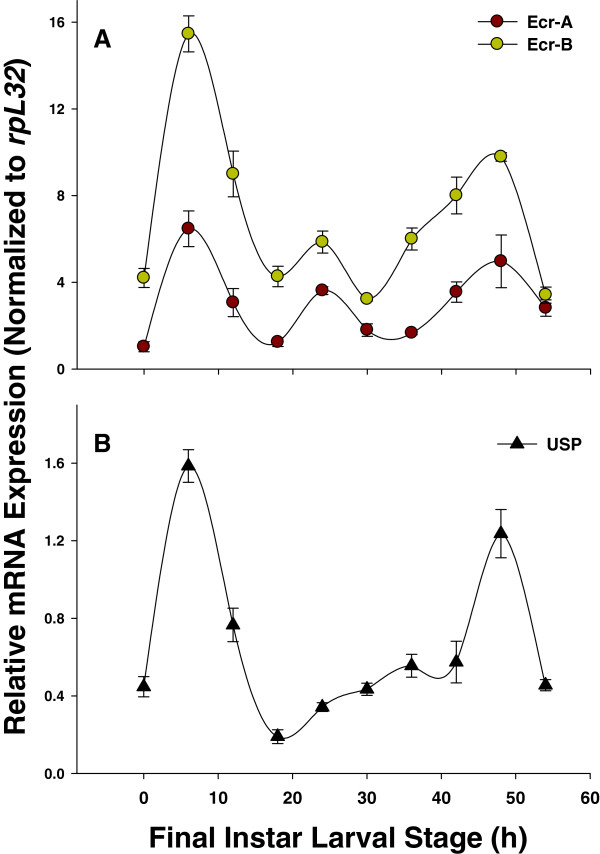
**Temporal expression profiles of PxEcRs (A) and PxUSP (B), respectively, in the last larval instar.** Insects were collected at a 6 h interval from the beginning of the 4th larval instar. The mRNA levels were examined by qRT-PCR and normalized to the ribosomal protein *rpL32* of *P. xylostella* (reference gene). Mean ± S.E. represents three independent replications.

**Figure 8:**
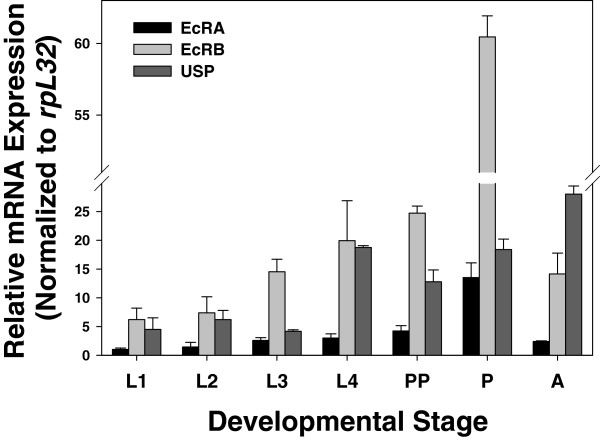
**The expression profiles of PxEcRs and PxUSP, respectively, during *P. xylostella *development.** Sample collections are detailed in Method section. The mRNA levels were determined by qRT-PCR and normalized to *rpL32* reference gene expression. Mean±S.E. represents three independent replications. L1-L4: 1^st^ to 4^th^ larval instar, PP: pre-pupae, P: pupae, A: adult.

In the final instar larval stage, PxEcRB mRNA was present at a high level with two peaks (Figure 7), one at the start of the final instar (6 h after ecdysis into the final instar) and the other at the end (48 h after ecdysis into the final instar, i.e., wandering stage). For PxEcRA, though the expression profile was similar to that of PxEcRB, the expression levels were relatively lower than those of PxEcRB (Figure 7). During the entire developmental stages, PxEcRB exhibited a significantly higher expression level, which was about 6-fold higher than that of PxEcRA. Nevertheless, the expression patterns of PxEcRA and PxEcRB were similar, and both of their expressions peaked in the pupal stage. For PxUSP, the expression pattern seemed to be ubiquitous throughout the last larval instar and the entire developmental stages. In the final larval instar, the two expression peaks of PxUSP coincided with those of the PxEcR isoforms, however, at other time intervals, the expression of PxUSP varied (Figure 7). Among the entire developmental stages, PxUSP peaked at the adult stage, which was different from PxEcRs (Figure 8).

## Discussion

In this study, we cloned and expressed the full length cDNAs of three ecdysone receptors including two EcR isoforms and one USP from *P. xylostella*. PxEcRA was obtained from the adult female, whereas, PxEcRB was readily obtainable in each and every developmental stage. We also determined the expression profiles of their respective mRNAs in the 4^th^ instar larvae as well as the entire developmental stages. Furthermore, when co-expressed with USP, the binding affinities of these EcR isoforms were examined.

Two EcR isoforms, PxEcRA and PxEcRB, differ in their A/B domain. These two isoforms belong to the Type 2 A and Type 6 B1 isoform, respectively, according to Watanabe *et al.*[30]. The N-terminal A/B region of EcRA and EcRB is isoform-specific, and might be an essential structural basis for the transcriptional activation functions [2,30,31]. The (K/R)RRW motif of the EcR-B1 isoform provides additional interaction sites for co-regulatory proteins and mediates the regulation of the B1 isoform-specific AF1 transactivation function [30]. In this study, for both PxEcRA and PxEcRB, two splicing variants with a 15 bp difference in the hinge domain were identified. A 15bp difference has also been reported in the splicing variants from *M. sexta*[3], *C. suppressalis*[20], and *Leptinotarsa decemlineata*[32]. However, the functional significance of these structural differences between the two variants has yet been fully characterized. In *C. suppressalis*, however, the lack of five amino acids (encoded by 15 nt) in the middle of D region of EcR did not affect the ligand-receptor binding [27]. Similar to EcRs, multiple USP isoforms have been found in lepidopteran *M. sexta* and *H. armigera*[33,34], dipteran *Aedes aegypti* and *Chironomus tentans*[35,36], and coleopteran *Tribolium castaneum*[37]. In this study, however, only one USP isoform was obtained in *P. xylostella*. Other insect species containing only one USP isoform includes *D. melanogaster*[38], *Chilo suppressalis*[39] and *Choristoneura fumiferana*[40].

Results from the ligand-binding assay demonstrated that 1) tritiated PonA specifically bound to PxEcRB but not PxUSP and PxEcRA, and the specific binding to PxEcRB was enhanced by the addition of PxUSP. This is consistent with previous observations in *C. suppressalis*[27], *L. decemlineata*[32], *C. tentans*[36,41] and *D. melanogaster*[42]; and 2) the presence of PxUSP could not enhance the binding of tritiated PonA to PxEcRA. Similar result was observed in the Dwarf Wood scorpion*, Liocheles australasiae*[43], where the retinoid X receptor did not enhance the binding of tritiated PonA to *L. australasiae* ecdysone receptor-A. Unlike *C. fumiferana*[44], where the two isoform complexes have similar binding affinities for ponasternone A, there is a distinct binding affinity between PxEcRA/USP and PxEcRB/USP.

The dissociation constant (*K*_d_ =3.0±1.7 nM) for the PxEcRB/USP heterodimer is comparable to EcR/USP complex from other insects, such as in lepidopteran 1.0-2.0 nM [27,44,45], coleopteran 2.8 nM for *L. decemlineata*[32], dipsteran 0.9-2.8nM [2,46], pentatomomor-phan 6.8 nM for *Nezara viridula*[47], and orthopteran 1.2 nM for *Locusta migratoria*[48]. Comparative analysis of receptor-binding affinity among insects sheds light on the divergence of the toxicity of ecdysone agonists.

Previous studies showed that the expression profiles of different isoforms vary among developmental stages and tissues. In *D. melanogaster*, EcRA isoform predominately locates at the imaginal discs, imaginal rings, and in two sets of specialized larval cells (the prothoracic gland cells). In addition, the EcRA isoform is associated with the onset of new cuticle synthesis in every molt so its presence is not metamorphic-specific. In contrast, the EcRB1 isoform is found primarily in larval tissues and in the imaginal histoblasts that form the abdominal epithelium and the midgut of adult [49,50]. Phenotypic analysis of mutants suggests that EcRA is required for pupal development [51] and EcRB1 is necessary for normal metamorphic development [49]. In *Bombyx mori*, during the larval-pupal transition, EcRB1 was broadly distributed in most tissues examined including midgut, epidermis, fat body, and the wing imaginal disc, while EcRA was found only in the anterior silk gland [52]. In this study, mRNA expression levels of PxEcRB were consistently 6-fold higher than that of PxEcRA in *P. xylostella* throughout the entire developmental stages. By contrast, the expression of EcRA in *L. decemlineata* was significantly higher than that of EcRB1, and EcRA was predominantly found in larval tissues [32]. The apparent spatial and temporal distribution discrepancies of EcR isoforms are likely due to the different species.

## Conclusion

This paper reports the molecular cloning, heterologous protein expression, and characterization of the three ecdysone receptors including two EcR isoforms and one USP from *P. xylostella*. As the target site of the DAH-based ecdysone agonist/molt-accelerating biopesticides, a comprehensive understanding of ecdysone receptors will shed light on the mechanistic study of DAH resistance in *P. xylostella*. The mode of action of this group of biopesticides is the induction of premature and lethal molting in insects [46]. One of the resistance mechanisms involves altered binding of ecdysone agonists to the ecdysone receptor [53,54], suggesting that target site insensitivity is the underlying resistance mechanism. Given the distinct differences at expression level and the ligand-binding capacity, we hypothesis that PxEcRB is the ecdysone receptor that controls the remodeling events during metamorphosis, and more importantly, the potential role played by PxEcRB in diacylhydrazine resistance in *P. xylostella* warrants further investigation.

## Abbreviations

EcR: Ecdysone receptor; USP: Ultraspiracle; 20E: 20-hydroxyecdysone; NRs: Nuclear receptor superfamily; AF-1: Activation function 1; DAH: Diacylhydrazine; JH: Juvenile hormone; DBD: DNA-binding domain; LBD: Ligand-binding domain; NLS: Nuclear localization signal.

## Competing interests

The authors have declared that no competing interest exists.

## Authors’ contributions

Conceived and designed the experiments: PL. Performed the experiments: BZT, WD. Analyzed the data: BZT. Wrote the paper: BZT, PL, XGZ. Contributed reagents/materials: XWG. All authors read and approved the final manuscript.

## Supplementary Material

Additional file 1 **Table S1. **Primers used in the study. Note: The sequences underlined at the 5’end are used for the directional cloning for *in vitro* expression and are not part of the receptor sequences. Click here for file

Additional file 2 **Figure S1. **Primary structure of *P. xylostella* EcR isoforms. **(A)** Nucleotide and deduced amino acid sequences of PxEcRB. Amino acid sequence is shown below the nucleotide sequence. The DNA binding domain (C region) is underlined, and the ligand binding domain (E region) is underlined with dashes. The five amino acids that is absent in some cDNAs is boxed. The putative P-box and D-box are shaded. The junction between PxEcRA and PxEcRB is shown by an arrow. The sequences in wavy line denote the putative nuclear localization signal (NLS), corresponding to the putative NLS of LXRα [55]. **(B)** The nucleotide and deduced amino acid sequences of the isoform-specific region of PxEcRA. Gly_117_ and rest of the downstream sequences are shared by both isoforms, and therefore, this common region was not presented in B. Click here for file

Additional file 3 **Figure S2. **Nucleotide and deduced amino acid sequence of *P. xylostella* USP***.*** PxUSP amino acid sequence is shown below the nucleotide sequence. The DNA binding domain (C region) is underlined, and the ligand binding domain (E region) is underlined with dashes. The 13 amino acids motif conserved in all USPs located upstream of the DBD is boxed. The putative P-box and D-box are shaded. The putative nuclear localization signal (NLS), corresponding to the putative NLS of *D. melanogaster*[1], is highlighted in bold and underlined. Click here for file

Additional file 4 **Figure S3. **Phylogenetic analysis of insect ecdysone receptors. The phylogenetic relationships of *P. xylostella* ecdysone receptors, including EcRA **(A)**, EcRB **(B)** and USP **(C)**, with other insects were inferred using the ML (maximum likelihood) estimation (Jones-Taylor-Thornton model, 500 bootstrap replicates). Click here for file

Additional file 5 **Table S2. **Comparison of amino acid identities of EcR isoforms and USP between *P. xylostella* and other insect species (%). Click here for file
