# Supplementary material for: Cloning, ligand-binding, and temporal expression of ecdysteroid receptors in the diamondback moth, Plutella xylostella
Source: BMC Mol Biol. 2012 Oct 19;13:32. doi: 10.1186/1471-2199-13-32 (PMC3568735; doi:10.1186/1471-2199-13-32)
Supplement: Additional file 1 — Table S1. Primers used in the study. Note: The sequences underlined at the 5’end are used for the directional cloning for in vitro expression and are not part of the receptor sequences. [file 1471-2199-13-32-S1.docx]

**Table S1 Primers used in the study**

| Primer | | Sequence (5’-3’) |
| --- | --- | --- |
| **ID** | **Purpose** |  |
| F1B | RT-PCR | GTGTGTTTCTGGCATTAGTTGG (for EcR-B) |
| R1B | RT-PCR | GTCTATGAGCGTCTCTCCTTGT |
| F1U | RT-PCR | CGGAGATGAAGCCAGACA (for USP) |
| R1U | RT-PCR | GGTGGAAGAAGAACAGDTGC |
| ER1 | 5’-RACE | GGCACTCCTGGCACTTTCTCCGCAT (for EcR-A) |
| UR1 | 5’-RACE | CCCATAGTGCTTCCCCGACGCCCTG (for USP) |
| UF1 | 3’-RACE | GTATTCCTGTCTGGACGAGT (for USP) |
| EAF | ORF | GGCTGCGATCGCCATGGACCTCAAGCAGGTGGAGGTG |
| EAR | ORF | TTGTGTTTAAACGAGCTCGGGGGCGTCGAGGG |
| EBF | ORF | AGGAGCGATCGCCATGAATCGTGGATTGTTTGACGGGCCCGCGACC |
| EBR | ORF | TTGTGTTTAAACGAGCTCGGGGGCGTCGAGGG |
| USPF | ORF | GGGTGCGATCGCCATGGAGCCCGGAAGAGAAGC |
| USPR | ORF | TGGTGTTTAAACCATCATAGAGTTAGTATCTATGGTCTGCACG |
| QEAF | qRT-PCR | CACCTGCGGGAGTGGATG |
| QEAR | qRT-PCR | GCTCGTGGACAGCGGTGAC |
| QEBF | qRT-PCR | GGAGAGTTCCTCGGAGGTGT |
| QEBR | qRT-PCR | TGCTGTAGGTGATGGAGTCG |
| QUF | qRT-PCR | CCTGGAGGGCGGGTTCAT |
| QUR | qRT-PCR | GGGTGGTTCGGGGGGTAG |

Note: The sequences underlined at the 5’end are used for the directional cloning for *in vitro* expression and are not part of the receptor sequences.
