# Supplementary material for: Cloning, ligand-binding, and temporal expression of ecdysteroid receptors in the diamondback moth, Plutella xylostella
Source: BMC Mol Biol. 2012 Oct 19;13:32. doi: 10.1186/1471-2199-13-32 (PMC3568735; doi:10.1186/1471-2199-13-32)
Supplement: Additional file 2 — Figure S1. Primary structure of P. xylostella EcR isoforms. (A) Nucleotide and deduced amino acid sequences of PxEcRB. Amino acid sequence is shown below the nucleotide sequence. The DNA binding domain (C region) is underlined, and the ligand binding domain (E region) is underlined with dashes. The five amino acids that is absent in some cDNAs is boxed. The putative P-box and D-box are shaded. The junction between PxEcRA and PxEcRB is shown by an arrow. The sequences in wavy line denote the putative nuclear localization signal (NLS), corresponding to the putative NLS of LXRα [55]. (B) The nucleotide and deduced amino acid sequences of the isoform-specific region of PxEcRA. Gly117 and rest of the downstream sequences are shared by both isoforms, and therefore, this common region was not presented in B. [file 1471-2199-13-32-S2.docx]

**A**

1 gactccgcaggcggcgaccgcgagcgctgaacgtgcgttcgaaccactctccgcgataaa

61 caacttcgcgtcgcgaattccgagcaacaagagttttgtttgtcgcgttatttatcctgt

121 cgaacaggttgtgtaattgtgatttttgcagtcgatttcgtttcggagtttccactttct

181 gaatctgttggtacgtgaacggacgaagtgcatctcaggcggattgcggagcgattagtt

241 tcggtttctgtgcggtgaccgtgtgtgtgtttctggcattagttggtgagaaagagtgtt

301 tgtgaaaggcgccaaatgccattcattt**ATG**AATCGTGGATTGTTTGACGGGCCCGCGAC

***1*** M N R G L F D G P A T

361 CATGAAACGCCACTGGTCCAACAACGGGGGGTTCCAGACTCTGCGCATGCTGGAGGAGAG

***12*** M K R H W S N N G G F Q T L R M L E E S

421 TTCCTCGGAGGTGTCCTCGTCGTCGGCGCTGGGCCTGCCGCCCGCCATGGTGCTCACGCC

***32*** S S E V S S S S A L G L P P A M V L T P

481 GGAGTCGCTGGCCTCGCCCGAGTACGGCGGCCTGGACCTGTGGGGCTACGACGACTCCAT

***52*** E S L A S P E Y G G L D L W G Y D D S I

541 CACCTACAGCACGGCGCAGACGCTGCTCAGCTCGGGCTGCACGCCGCCGCAGCCCAAGCC

***72*** T Y S T A Q T L L S S G C T P P Q P K P

601 GCCGCACGCGCTGCYCTCCATGCCTCTCCCCATGCCGCCCACCACGCCCAAATCGGAGAA

***92*** P H A L X S M P L P M P P T T P K S E N

661 CGAATCCATCTCATCAGGCCGCGAGGAGCTCTCSCCAGCGTCCAGTGTGAACGGCTGCAG

***112*** E S I S S G R E E L S P A S S V N G C S

721 CAATGACGGCGACGCGCGKAGGCCCAAGAAGGGCCCGGCGCCGCGGCARCAGGAGGAGCT

***132*** N D G D A R R P K K G P A P R Q Q E E L

781 GTGCCTGGTCTGCGGGGACCGGGCCTCGGGGTACCACTACAACGCGCTCACGTGTGARGG

***152*** C L V C G D R A S G Y H Y N A L T C E G

841 ATGCAAAGGATTCTTCAGGCGGAGCGTCACAAAGAACGCAGTCTACATCTGCAAGTTCGG

***172*** C K G F F R R S V T K N A V Y I C K F G

901 GCACGCGTGCGAAATGGACATGTACATGCGGAGAAAGTGCCAGGAGTGCCGGCTGAAGAA

***192*** H A C E M D M Y M R R K C Q E C R L K K

961 GTGCCTCGCCGTGGGCATGAGGCCCGAGTGTGTGGTACCGGAGACTCAGTGCGCGATAAA

***212*** C L A V G M R P E C V V P E T Q C A I K

1021 GAGGGAAGAGAAGAAGGCACAAAGGGAGAAGGATAAGCTGCCTGTTAGTACGACGACGGT

***232*** R E E K K A Q R E K D K L P V S T T T V

1081 GGATGATCATATGCCCCCGATCATGCAGTGCGAGCCGCCGCCCCCTGAAGCCGCTAGGAT

***252*** D D H M P P I M Q C E P P P P E A A R I

1141 TCTCGACTGTTTGCAGCACGAAGTGGTCCCGCGGTTCCTGTCGGACAAGCTGCTGGAGCA

***272*** L D C L Q H E V V P R F L S D K L L E Q

1201 GAACCGAGCCAAGAACATCCCCCCCCTCACGCCCAACCAGAAGTTCCTCATAGCCAGGCT

***292*** N R A K N I P P L T P N Q K F L I A R L

1261 CGTCTGGTACCAGGACGGGTACGAGCAACCCTCGGAGGAAGACCTGAAGCGGGTCACGCA

***312*** V W Y Q D G Y E Q P S E E D L K R V T Q

1321 GACGTGGCAAGCGTCAGACGACGACGAAGAAGAGTCGGACATGCCGTTCCGGCAGATCAC

***332*** T W Q A S D D D E E E S D M P F R Q I T

1381 GGAGATGACCATCCTCACCGTGCAGCTCATCGTGGAGTTCGCGAAGGGGCTGCCCGGCTT

***352*** E M T I L T V Q L I V E F A K G L P G F

1441 CGCCAAGATCTCGCAACCGGATCAGATCACGTTACTGAAGGCGTGTTCAAGCGAGGTGAT

***372*** A K I S Q P D Q I T L L K A C S S E V M

1501 GATGCTGCGAGTGGTACGGCGGTATGACGTCACCACAGACAGCATCCTGTTCGCCAACAA

***392*** M L R V V R R Y D V T T D S I L F A N N

1561 CCAGGCGTACACTCGAGACAACTACCGCAAGGCAGGCATGGCGTACGTCATCGAGGACTT

***412*** Q A Y T R D N Y R K A G M A Y V I E D L

1621 GCTGCACTTCTGTCGCTGCATGTACTCGCTGAATATGGACAATATACACTATGCGCTGTT

***432***  L H F C R C M Y S L N M D N I H Y A L L

1681 GACGGCGATTGTTATATTCTCAGACCGGCCGGGGCTGGAGCAGCCTCAACTAGTGGAAGA

***452*** T A I V I F S D R P G L E Q P Q L V E E

1741 GATCCAGAAGTACTACCTGAACACTCTCCGGGTATACATCCTGAGCCAGCAGAGCGGCTC

***472*** I Q K Y Y L N T L R V Y I L S Q Q S G S

1801 GCCCAAGTGCCCCGTGATCTTCGGCTGGATCCTGTCCATCCTCTCCGAACTGCGCTCGCT

***492***  P K C P V I F G W I L S I L S E L R S L

1861 CGGCACGCAGAACTCCAACATGTGCATCTCACTCAAGCTCAAGAACAAGAAGCTGCCCCC

***512***  G T Q N S N M C I S L K L K N K K L P P

1921 CTTCCTCGAGGAGATCTGGGACGTGGCCGACGTGTCCACTCCCGCCCCCGCCGCCGCCCC

***532*** F L E E I W D V A D V S T P A P A A A P

1981 CCTCGACGCCCCCGAGCTCTAGcccgcgcgccccctctaacgcacggctcacaaggagag

***552***  L D A P E L *

2041 acgctcatagactggctagttttagtgaagtgcgacagacctatacggattgtgcaaatt

2101 tatgtatattcgacgagatcacgtctcgcgttcgggtgggtatatgtgcgagagattagt

2161 gaatatatgtgttgttgaacgtttggagaatatatatttagtgttgattgtttacggccg

2221 aggccgagggccggtggccgcgactgtaccgcgcacgtgcgcgactgtacaggactgcag

2281 ccggtagctacctacgcatcctatgaaaccatacgtacactacgagagttatacaaaaaa

2341 aaaaaaaaaaaaaacaaa

**B**

1 agtcggcggcggcgagtcgcgcgcgtcgtcccctatcacttgtctcccgagctcgttgcg

61 ctacgctctcaggctgccgctcctcagccctgctgctctcacgctccggagcgtggaggt

121 cgctgcgacctcgccgagccggcctctcggtttcaccgcgccgactgactgtcgttcacc

181 cagtgtcgctgcaggcgaaatactcttgaaccgggtctgtggtcttgtgctgtaatgccg

241 acggggcagtcggctgatccgtgttgtgacagtgaaatcgtgagccttgcccgtgcggat

301 gtgcagtgatggataaatagacgaggagtagagtgttgtgaccgtgcaccaggaggcgga

361 gggttttttgagaaagtgagagaacgagtcgtcaaaattgagcctaaattgtatatagat

421 tacaacgccgcccctctgttcgcctcggaaaacgcctccagtgtgtggggctcgggcggc

481 ggcggcggcggcgcagcatgcggtaacagcggc**ATG**GACCTCAAGCAGGTGGAGGTGGCG

***1*** M D L K Q V E V A

541 TACCGCGGGCTGCCCGGCGTGAAGGCCGAGCCGGGGGTCTCCCACGGGCACCAGCCGCTC

***10*** Y R G L P G V K A E P G V S H G H Q P L

601 AACGGGCACCTGCGGGAGTGGATGGGGGGCGGAGCCGACTCCCCATCCCCCGGAGCCGCC

***30*** N G H L R E W M G G G A D S P S P G A A

661 GCGCAGCCGCAGAGCAATGGGTACTCGTCACCGCTGTCCACGAGCAGCTATGGGCCGTAT

***50*** A Q P Q S N G Y S S P L S T S S Y G P Y

721 AGTCCGAATGGGAAAATA

***70*** S P N G K I

**Figure S1. Structure of *P. xylostella* EcR (PxEcR) isforms.** **(A)** Nucleotide and deduced amino acid sequence of PxEcRB. Amino acid sequence is shown below the nucleotide sequence. The DNA binding domain (C region) is underlined, and the ligand binding domain (E region) is underlined with dashes. The five amino acids that is absent in some cDNAs is boxed (the same situation exists in PxEcRA). The putative P-box and D-box are shaded. The junction between PxEcRA and PxEcRB is shown by an arrow. Gly_117_ and the downstream sequences were omitted in (B) for they are common between PxEcRA and PxEcRB. The sequences in wavy line denote the putative nuclear localization signal (NLS), corresponding to the putative NLS of LXRα [[56](#_ENREF_56)]. **(B)** The nucleotide and deduced amino acid sequence of the isoform-specific region of PxEcRA.
