# Supplementary material for: Cloning, ligand-binding, and temporal expression of ecdysteroid receptors in the diamondback moth, Plutella xylostella
Source: BMC Mol Biol. 2012 Oct 19;13:32. doi: 10.1186/1471-2199-13-32 (PMC3568735; doi:10.1186/1471-2199-13-32)
Supplement: Additional file 3 — Figure S2. Nucleotide and deduced amino acid sequence of P. xylostella USP. PxUSP amino acid sequence is shown below the nucleotide sequence. The DNA binding domain (C region) is underlined, and the ligand binding domain (E region) is underlined with dashes. The 13 amino acids motif conserved in all USPs located upstream of the DBD is boxed. The putative P-box and D-box are shaded. The putative nuclear localization signal (NLS), corresponding to the putative NLS of D. melanogaster[1], is highlighted in bold and underlined. [file 1471-2199-13-32-S3.docx]

1 tatgtacctatgtgtgtgtgtacggaaataatacttttgtgtattagaaatttaaagttt

61 tcaatttgaaaggctttggtgttgaaataagacagttttaagttgtttctaatttaattc

121 acagtgtttctatcagctatatgacagtgcaaaagattgtggtttctgttataggtatat

181 ttagttcaacgaccttgttactgacagtctgacaggttgatcgttgtggtgactgaacta

241 atattgcatgaaagtggtgcttctaccgtaaatacaagaaccctggaggcg**ATG**GAGCCC

***1*** M E P

301 GGAAGAGAAGCAGGGCTGAACCTGGAGGGCGGGTTCATGTCGCCGATGTCCCCGCCGGAG

***4*** G R E A G L N L E G G F M S P M S P P E

361 ATGAAGCCAGACACGGCCATGCTGGACGGGCTGCGAGACGACGCCACCTCGCCCCCCGCG

***24*** M K P D T A M L D G L R D D A T S P P A

421 TACCGCAACTACCCCCCGAACCACCCGCTCAGCGGCTCCAAGCACCTGTGCTCGATATGC

***44*** Y R N Y P P N H P L S G S K H L C S I C

481 GGGGACAGGGCGTCGGGGAAGCACTATGGGGTTTATAGTTGCGAGGGCTGCAAGGGCTTC

***64*** G D R A S G K H Y G V Y S C E G C K G F

541 TTCAAGCGGACGGTGCGCAAGGACCTGACCTACGCTTGCCGCGAAGAGCGCAACTGCATC

***84*** F K R T V R K D L T Y A C R E E R N C I

601 ATCGACAAGCGGCAGAGGAACCGCTGCCAGTACTGCCGCTACCAGAAGTGCCTCGCGTGC

***104*** I  **D K R Q R N R C Q**  Y C R Y Q K C L A C

661 GGCATGAAGCGCGAGGCGGTGCAGGAGGAGCGGCAGCGGGCCGCGCGCGGCGCCGAGGAC

***124***  G M K R E A V Q E E R Q R A A R G A E D

721 GCGCACCCCAGCAGCTCTATACAGATCGTGCTACAGGAGCTGTCGATCGAGCGCCTCCTG

***144***  A H P S S S I Q I V L Q E L S I E R L L

781 GAAATGGAGTCGCTGGTGGCGGACCCGAGCGAGGAGTTCCAGTTCCTGCGCGTGGGGCCG

***164***  E M E S L V A D P S E E F Q F L R V G P

841 GACAGCAACGTGCCGCCGCGGTACCGCGCGCCCGTCTCCAGCCTGTGCCAGATCGGCAAC

***184*** D S N V P P R Y R A P V S S L C Q I G N

901 AAGCAGATCGCGGCGTTGGTGGTGTGGGCGCGCGACATCCCGCACTTCAGCCAACTGGAG

***204*** K Q I A A L V V W A R D I P H F S Q L E

961 CTGGATGACCAGGTGGTGCTCATCAAGAGCTGCTGGAACGAGCTGCTGCTGTTCGCTATA

***224***  L D D Q V V L I K S C W N E L L L F A I

1021 GCCTGGCGCTCTATGGAGTACCTGGAGGACGAGCGTGAGAACGCGGACGGGTCGCGCAGC

***244***  A W R S M E Y L E D E R E N A D G S R S

1081 TCCGCGCCGCCGCAACTCATGTGCCTAATGCCAGGCATGACGCTGCACCGCAACTCGGCG

***264*** S A P P Q L M C L M P G M T L H R N S A

1141 CTGCTGGCGGGCGTGGGGCAGATCTTCGACCGCGTGCTGTCGGAGCTCAGCATGAAGATG

***284*** L L A G V G Q I F D R V L S E L S M K M

1201 CGCGCGCTGCGCATGGACCAGGCCGAGTACGTGGCGCTCAAGGCGATCATACTGCTCAAC

***304*** R A L R M D Q A E Y V A L K A I I L L N

1261 CCAGATGTTAAAGGACTGAAAAACCGACTAGAAGTCGAAGCATTACGGGAAAAGATGTAT

***324***  P D V K G L K N R L E V E A L R E K M Y

1321 TCCTGTCTGGACGAGTACTGTCGGCGGTCGCGCAGCAGCGAGGAGGGCCGCTTCGCCTCG

***344*** S C L D E Y C R R S R S S E E G R F A S

1381 CTCCTCCTCCGCCTGCCCGCCTTACGCTCCATCTCGCTCAAGAGCTTCGAGCACCTGTTC

***364*** L L L R L P A L R S I S L K S F E H L F

1441 TTCTTCCATCTCGTGGCGGACTCCAGCATCGCTAGTTATATCAAGGACGCGTTAAGGAGC

***384*** F F H L V A D S S I A S Y I K D A L R S

1501 CACGTGCAGACCATAGATACTAACTCTATGATGTAGgtctcgcttgtttgtgtgcgcagt

***404*** H V Q T I D T N S M M *

1561 gctgtaacctcgggtttggtgggtgatttgcactcacttttgagctctcagtagagctag

1621 ttggtgcaaattggccgtgccgcgccgtataagttgcgtgatgcattttatttgatcaaa

1681 tagagtgaaagctatccgagatgaatagtaggtacataaatatataaaatgtctatttcg

1741 tcagatgagatttatcaagcatccatacctatagaagatgaacgcggcggcgtcaaaaac

1801 cgaacagaggttacaatgcacgaattataataatgtttcaatcaatagatcagtagtgtt

1861 taactacgtctatactaattgtagctcctataaaccttctggtacaattttaaccctctt

1921 ctagtaggaattgattgaaactaatgttatcgatgtctagtgcgtaaaagcgaggctaac

1981 actgtccctacattttctgactgaacctacagtttcctccatcgatgcaatatgctgaac

2041 aaaacagagcccctttttgagagatggattcatgtgaattatgtaatggaaaagtaaata

2101 atattaaagatattttgaccgacactataggatcgctggtcgatacttgtctaaaaatat

2161 atttaaaagtcttggatcaaaatatagtgtgaatatcagatgttagatattttaaatggg

2221 gccgcctctttttattaatattacgtagatatattacattaatgtaattaacgtaagttg

2281 ttcatctattagtgaattttatccgttaagtagtgtctatggtatattaattaataatac

2341 atattacaactatttttattactgtgtaaattaacatttaccaaatctctcttttaatta

2401 ttcgacattgaaaatgtccaaagtgccctcaacgttgttgactgcaaatataatcttgtc

2461 tattcgagaatcttctaattaaatctaaaattgttaaattatgtctccgctgcatattct

2521 attcacatttgtggtgctttaaatacataatgtagatatgcagctacaagtatcaagatt

2581 ctgcccgtatacctgaaacctatacgctattcataatgtttgtaaatagttatacctaag

2641 tatgtactattattgctgttagatcttattttaaggaacctagcctcaccagttgttggt

2701 gatggggtgaagaaggcggttaacgaatggcctatgaaataacgttatttacattgtaat

2761 ctgcatggccagtcgactagtgaaacctcattactaaaatgcatttccgagaccagtaag

2821 taaccagtattaaaatgtttaagacgaaaatttataacgcccgaaaagaataacgaatgc

2881 ttcaaagacggttatttaatatgattgaaaaataataatgtttgtagatcgtacctgcat

2941 gtgatgtaactgaatgttgtattttacaaataatatattttgattcgacggtgagtgttg

3001 tggaaggttcatttgattagttaattgttgtaaaagttgcatttgaagcggtcaaaccaa

3061 aaatattcgtgattgaagcttctacagcattcagtttgattcctctcactgtatctttca

3121 cacaatttaacctgagggttaagttctagttattcgactgccgaaacaccatactaaatt

3181 ttgtcaggctctgagcaaatatgcgtaacaaaatttaaatttttgacattgatgaattgt

3241 tatcttttcattggtttattttcgagaaactcgcactaaatacgcgataattaatttgtg

3301 tgaaagattcagtgagtgttcgcattgtagagaagacgtagagtttaccggtgatgggat

3361 gctcacgactgccaaagcagaggttcttgggatccccctgggtacagaatatgttgagtt

3421 ggatataatgttattattctatgaatgggtatgaatctgttatagaggtgttgcagaggt

3481 tgtgagatgatttataatgacgtttactaattaaaatctgactatgtattctaagttata

3541 ttttttgtatagtaatagttataagttgaatggtaaataaaaatattatcatcacaaaaa

3601 aaaaaaa

**Figure S2. Nucleotide and deduced amino acid sequence of USP of *P. xylostella.*** PxUSP amino acid sequence is shown below the nucleotide sequence. The DNA binding domain (C region) is underlined, and the ligand binding domain (E region) is underlined with dashes. The 13 amino acids motif conserved in all USPs located upstream from the DBD is boxed. The putative P-box and D-box are shaded. The sequences in large bold font and large bold underline denote the putative nuclear localization signal (NLS), corresponding to the putative NLS of *D. melanogaster* [[1](#_ENREF_1)].
