# Supplementary material for: Cloning, ligand-binding, and temporal expression of ecdysteroid receptors in the diamondback moth, Plutella xylostella
Source: BMC Mol Biol. 2012 Oct 19;13:32. doi: 10.1186/1471-2199-13-32 (PMC3568735; doi:10.1186/1471-2199-13-32)
Supplement: Additional file 4 — Figure S3. Phylogenetic analysis of insect ecdysone receptors. The phylogenetic relationships of P. xylostella ecdysone receptors, including EcRA (A), EcRB (B) and USP (C), with other insects were inferred using the ML (maximum likelihood) estimation (Jones-Taylor-Thornton model, 500 bootstrap replicates). [file 1471-2199-13-32-S4.docx]

**Blattaria**

**Lepidoptera**

**Coleoptera**

**Diptera**

**Hymenoptera**

*P.xylostella EcRA*

*P.xylostella EcRB*

**Coleoptera**

**Lepidoptera**

**Diptera**

**Coleoptera**

*P.xylostella USP*

**Lepidoptera**

**Hymenoptera**

**Diptera**

**A**

**B**

**C**

**Figure S3.**
