# Supplementary material for: Cloning, ligand-binding, and temporal expression of ecdysteroid receptors in the diamondback moth, Plutella xylostella
Source: BMC Mol Biol. 2012 Oct 19;13:32. doi: 10.1186/1471-2199-13-32 (PMC3568735; doi:10.1186/1471-2199-13-32)
Supplement: Additional file 5 — Table S2. Comparison of amino acid identities of EcR isoforms and USP between P. xylostella and other insect species (%). [file 1471-2199-13-32-S5.docx]

**Table S2. Comparison of amino acid identities of EcR isoforms and USP between *P. xylostella* and other insect species (%)**

| **Species**  **Species** | **EcRA**  **EcRA** | **EcRB**  **EcRB** | **USP**  **USP** |
| --- | --- | --- | --- |
| *Choristoneura fumiferana* | 87 | 86 | 89 |
| *Chilo suppressalis* | 86 | 87 | 88 |
| *Plodia interpunctella* | - | 84 | 87 |
| *Helicoverpa armigera* | - | 84 | 88 |
| *Heliothis virescens* | *-* | 85 | - |
| *Spodoptera frugiperda* | - | 83 | 88 |
| *Spodoptera littoralis* | - | 82 | 88 |
| *Spodoptera litura* | - | 85 | 88 |
| *Spodoptera exigua* | 88 | 78 | 88 |
| *Bombyx mori* | 77 | 80 | 83 |
| *Manduca sexta* | 70 | 86 | 88 |
| *Aedes aegypti* | 70 | 60 | 61 |
| *Ceratitis capitata* | - | 65 | - |
| *Drosophila melanogaster* | 66 | 64 | - |
| *Leptinotarsa decemlineata* | 60 | 57 | 51 |
| *Tribolium castaneum* | 65 | 57 | 71 |
